# The value of terahertz three-dimensional tomography for the differential diagnosis of tumor tissue and normal tissue in lung cancer nude mice

**DOI:** 10.3389/fbioe.2026.1923227

**Published:** 2026-09-18

**Authors:** Jin Zhu, Yang Li, Xiaoxue Zhang, Siqi Yin, Juntai Xu, Zhuang Yu, Shengnan Ren, Zhaoxin Guo, Dongwei Zhai, Chuan Li

**Affiliations:** 1 Qingdao Medical College, Qingdao University, Qingdao, Shandong, China; 2 Pingdingshan First People’s Hospital, Pingdingshan, Henan, China; 3 Oncology Department, The First Affiliated Hospital of Zhengzhou University, Zhengzhou, Henan, China; 4 Oncology Department, Qingdao Haici Hospital, Qingdao, Shandong, China; 5 Oncology Department, The Affiliated Hospital of Qingdao University, Qingdao, Shandong, China; 6 School of Physics Sciences, Qingdao University, Qingdao, Shandong, China; 7 Thoracic Surgery Department, The Affiliated Hospital of Qingdao University, Qingdao, Shandong, China

**Keywords:** gold nanorods, *in vivo* imaging, lung adenocarcinoma, lung squamous cell carcinoma, terahertz spectroscopy

## Abstract

**Introduction:**

To investigate the value of terahertz (THz) spectroscopy combined with gold nanorods (GNRs) in the differential diagnosis of lung squamous cell carcinoma (LUSC) and lung adenocarcinoma (LUAD).

**Methods:**

Tumor-bearing nude mouse models were established using GNR-treated A549 (LUAD) and NCI-H520 (LUSC) cells. Reflective THz 3D imaging and transmission THz time-domain spectroscopy (THz-TDS) systems, together with CT imaging and pathological validation, were employed to analyze the THz spectral characteristics of *in vivo* tumors, paraffin-embedded tissues, and thin sections.

**Results:**

CCK-8 and live/dead staining supported the biocompatibility of 40 μg/mL GNRs under the tested conditions. *In vivo* THz imaging showed higher tumor-region signals in the GNR-treated groups. At 0.5 THz, the model-specific tumor-to-adjacent skin/subcutaneous ROI contrast ratio in the LUSC group was 36:1 after GNR treatment and 22:1 in the untreated control; The contrast ratio of the GNR-treated LUSC group is 25:1, while that of the untreated control group is 19:1. Among the tested frequency bands, 0.5 THz provided the clearest image contrast. Contrast decreased markedly in paraffin-embedded tissues (approximately 2:1), and spectral differences in routine 5 μm human sections were obscured by the glass substrate. These findings support proof-of-concept signal enhancement in a subcutaneous xenograft model but do not establish a clinical tumor-to-normal-lung contrast ratio.

**Discussion:**

Under the tested conditions, GNR treatment was associated with higher THz signals in subcutaneous LUSC and LUAD xenografts. The mechanism and clinical performance remain unestablished; the approach should be regarded as proof-of-concept and as a potential complement to, rather than a replacement for, standard histopathology.

## 1 Introduction

Lung cancer is a leading cause of cancer-related mortality worldwide (Bray et al., 2024), rendering early diagnosis and personalized treatment enduring priorities in medical research. Lung cancer is broadly categorized into non-small cell lung cancer (NSCLC), accounting for approximately 80% of cases, and small cell lung cancer (20%) (Wang et al., 2022). Among these, lung adenocarcinoma (LUAD, ∼40%) and lung squamous cell carcinoma (LUSC, ∼30%) represent the two predominant histological subtypes of NSCLC (Yang et al., 2022). LUAD typically arises in the peripheral lung and exhibits a propensity for early metastasis, whereas LUSC is often centrally located in the bronchial airways and displays pronounced local invasiveness (Wang et al., 2022). Low-dose computed tomography (CT) has become the primary modality for lung cancer screening to minimize radiation exposure (García-Figueiras and Baleato-González, 2025). Nevertheless, patients with NSCLC remain highly susceptible to recurrence and mortality, as most are diagnosed with locoregional or distant metastasis. Both subtypes are characterized by high invasiveness and heterogeneity, capable of disrupting the lung parenchyma, invading the pleura, blood vessels, nerves, and mediastinum, leading to progressive respiratory impairment (Wang et al., 2023; de Koning et al., 2020). This destructive growth not only complicates therapeutic intervention but also severely compromises patient prognosis and quality of life (Luo et al., 2025). Consequently, there is an urgent need to develop novel tumor detection techniques for early NSCLC identification. This study investigates the potential of terahertz (THz) technology in the detection and discrimination of NSCLC.

Terahertz technology is a non-ionizing detection modality that is sensitive to tissue dielectric and structural properties (Sun et al., 2021), although strong water absorption can markedly attenuate signals from fresh or living tissue (Wang et al., 2024; Cong et al., 2023; Brown et al., 2010). Previous studies have evaluated THz spectroscopy or imaging in several tumor settings (Wang et al., 2025), including breast, gastric, colorectal, skin, brain (Oh et al., 2014), liver, cervical, and lung models (Ashworth et al., 2009; Grigorev et al., 2019; Chen et al., 2023; Joseph et al., 2014; Xu et al., 2025; Guan et al., 2023; Shi et al., 2021; Li et al., 2025). In the present study, GNRs were evaluated empirically as a candidate contrast agent; their near-infrared plasmonic properties do not by themselves imply a resonance mechanism in the THz band. A549 and NCI-H520 cells were used as established *in vitro* models of lung adenocarcinoma and lung squamous cell carcinoma, respectively (Meraz et al., 2025; Werner et al., 2023 Foster et al., 1998; Banks-Schlegel et al., 1985).

Based on this background, the present study utilized A549 and NCI-H520 cell line-derived tumor-bearing nude mouse models, employing reflective three-dimensional *in vivo* imaging combined with transmission-mode spectroscopic detection to systematically evaluate the diagnostic capability of THz technology within a dynamic tissue environment. This research aims to comprehensively and objectively assess the practical efficacy of THz technology for the early diagnosis of LUAD and LUSC, thereby providing a reliable experimental basis for its clinical translation.

## 2 Materials and methods

### 2.1 Materials

#### 2.1.1 Reagents

The human lung squamous cell carcinoma cell line NCI-H520 and the human lung adenocarcinoma cell line A549 were both purchased from Wuhan Boster Biological Technology Co., Ltd. (Wuhan, China). Fetal bovine serum (Pricella Biotech Co., Wuhan, China), DMEM basal medium (Pricella Biotech Co., Wuhan, China), penicillin-streptomycin-amphotericin B antibiotic mixture, trypsin, phosphate-buffered saline (PBS), and T75 cell culture flasks (Thermo Fisher, Shanghai, China) were used. Citrate-stabilized gold nanorods (GNRs) (Catalog No. XFJ24 7440-57-5, concentration: 0.1 mg/mL in water, absorption peak: ∼800 nm) were purchased from XFNANO Materials Tech Co., Ltd. (Jiangsu, China) and stored at 4 °C.

#### 2.1.2 Experimental animals

Twenty male nude mice, aged 4–6 weeks, were provided by Henan Skobes Biotechnology Co., Ltd. (Production License No.: SCXK (Yu) 2025–0005). No mice died during the experimental period, and all 20 mice, weighing 20–30 g, were included in the final analysis. The animals were housed in a specific pathogen-free (SPF) facility under controlled conditions: temperature (23 ± 2)°C, relative humidity (55 ± 5)%, with a 12-h light/dark cycle. Mice had free access to food and water. Sterilized SPF-grade bedding was used, and ultrapure water was provided for drinking. Food and water were replenished daily, and bedding was changed every 48 h. All cages and water bottles were disinfected with 84 disinfectant, rinsed with tap water, and autoclaved. This study was approved by the Animal Welfare and Ethics Committee of Qingdao University (Approval No.: 20250402BALB/C-nude2020251110246).

#### 2.1.3 Tissue samples

Tumor tissues and adjacent lung tissues were collected postoperatively from patients with lung cancer who were treated at the Laoshan Branch of The Affiliated Hospital of Qingdao University between March 2021 and April 2025. All patients were definitively diagnosed with lung squamous cell carcinoma or lung adenocarcinoma based on pathological examination. Written informed consent was obtained from all patients. This study was approved by the Ethics Committee of The Affiliated Hospital of Qingdao University.

#### 2.1.4 Instruments and equipment

The primary instruments used in this study included a reflective-mode terahertz (THz) three-dimensional tomography system and a transmission-mode THz time-domain spectroscopy (THz-TDS) system (QT-TO1000, Qingdao Qingyuanfengda Terahertz Technology Co., Ltd., Qingdao, China). *In vivo* micro-computed tomography (micro-CT) imaging was performed using a Quantum GX2 system (Japan). Digital slide scanning was conducted using a Pannoramic MIDI system (3DHISTECH, Hungary).

## 3 Experimental methods

### 3.1 Cell model preparation

GNR-treated NCI-H520 and A549 cell preparations were generated by incubating cells with GNRs. NCI-H520 cells were cultured in complete medium containing 10% fetal bovine serum (FBS) and 90% RPMI-1640, while A549 cells were cultured in complete medium containing 10% FBS and 90% DMEM basal medium. Penicillin-streptomycin-amphotericin B antibiotic mixture (1 mL) was added to every 100 mL of culture medium. Cells were grown in T75 culture flasks and maintained at 37 °C in a humidified atmosphere with 5% CO_2_. The culture medium was replaced every 48 h, and cells were passaged upon reaching optimal confluence. GNRs were added to the culture medium at 40 μg/mL, and the cells were incubated in the presence of poly-L-lysine (PLL) for 24 h. This protocol defines exposure to GNRs but does not establish whether the particles were internalized or remained cell-surface/extracellularly associated.

### 3.2 CCK-8 assay

Cells in the logarithmic growth phase were seeded into 96-well plates at a density of 1 × 10^3^ cells per well, with a total volume of 100 μL per well. The following day, after cell adherence and restoration of normal morphology, cells were exposed to the specified GNR concentrations. At 0, 24, 48, 72, and 96 h after GNR exposure, the culture medium was discarded and replaced with 90 μL of fresh complete medium and 10 μL of CCK-8 reagent per well. After gentle mixing, the plates were incubated for 1–4 h, and absorbance was measured at 450 nm using a microplate reader. Air bubbles were avoided during measurement to prevent interference with optical-density determination.

### 3.3 Live/Dead cell staining

Cells in the logarithmic growth phase were seeded into 24-well plates at a density of 1 × 10^5^ cells per well and cultured overnight. The culture medium was discarded, and cells were washed with PBS. Working solution containing calcein-AM (2–4 μM) and propidium iodide (4–8 μM) was added, followed by incubation in the dark for 20–30 min. Observation was performed under a fluorescence microscope, with 3–5 random fields per well photographed. Exposure parameters were maintained consistently across all images, and bright-field images were simultaneously captured to observe cell morphology.

### 3.4 Establishment of tumor-bearing nude mouse models

Upon reaching logarithmic growth phase, the culture medium was removed from each T75 flask, and 2 mL of trypsin was added.The flasks were incubated at 37 °C for 3 min for digestion. Following digestion, 2 mL of complete culture medium was added to terminate the digestion process. Cells were gently dissociated by pipetting, and the resulting cell suspension was transferred to 15 mL centrifuge tubes. Cells were centrifuged at 1,000 rpm for 5 min, and the supernatant was discarded. Cells were resuspended in 2 mL of complete culture medium, and cell concentration was determined using a cell counter. Twenty male nude mice (4–6 weeks old, SPF grade) were acclimatized for 3 days prior to the experiment. Approximately 1 × 10^7^ cells were injected subcutaneously into the right dorsal hind limb of each mouse. Ten nude mice were inoculated with NCI-H520 cells, and the remaining ten were inoculated with A549 cells. Tumor-bearing mice were randomly divided into experimental and control groups, with five nude mice in the experimental group receiving GNR-treated cells and five in the control group receiving non-GNR-treated cells. The entire procedure, from cell digestion to subcutaneous inoculation, was completed within 30 min. Approximately 3 weeks later, when tumors reached approximately 1 cm in diameter and all 20 nude mice exhibited stable vital signs, the models were deemed successfully established.

### 3.5 Terahertz 3D imaging and CT scanning

Following the establishment of subcutaneous tumor transplantation models in nude mice, further analysis was performed using THz and CT imaging. Three weeks after subcutaneous tumor inoculation, mice were anesthetized with 3% isoflurane inhalation, and imaging was performed using the 3D imaging mode of the THz system (Figure 1). Imaging conditions were as follows: temperature 25 °C, relative humidity 20%, frequency range 0.1–3.0 THz, scanning step size 200 μm, and ambient air. Imaging results were compared between experimental and control groups. Additionally, nude mice were anesthetized with 3% isoflurane, and *in vivo* micro-CT scanning was performed using a small animal CT system as a reference. As illustrated in Figure 2B, THz emission signals were focused onto the living mouse through a lens with a focal length of 10 cm, generating reflections. The spot diameter was less than 2 mm. THz waves carrying spectral information from the mouse passed through the lens to the THz signal detector. The specimen holder beneath the mouse enabled two-dimensional movement with a step size of 200 μm, and the imaging speed was 60 pixels/second.

**FIGURE 1:**
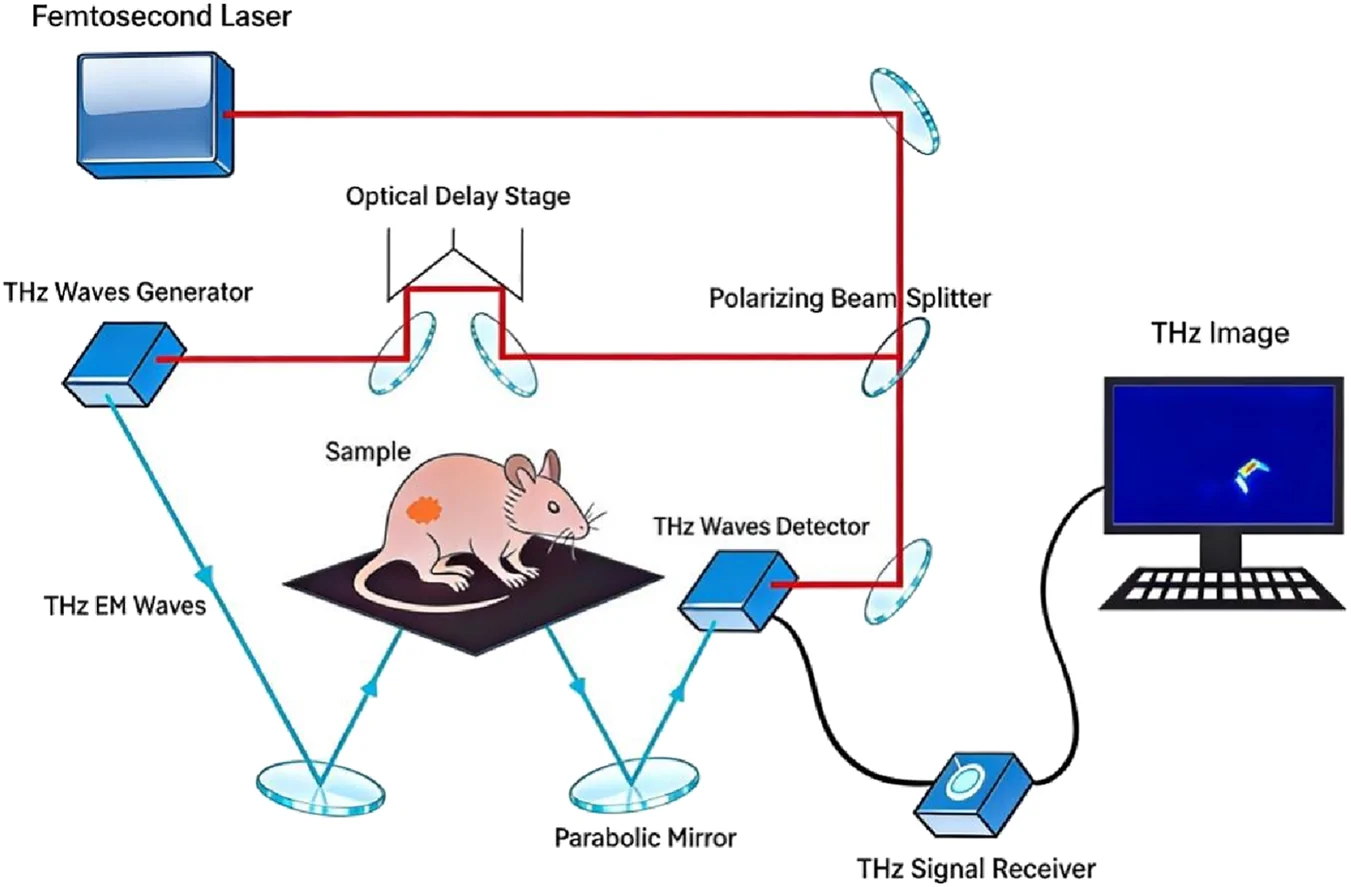
Schematic diagram of terahertz three-dimensional *in vivo* imaging.

**FIGURE 2:**
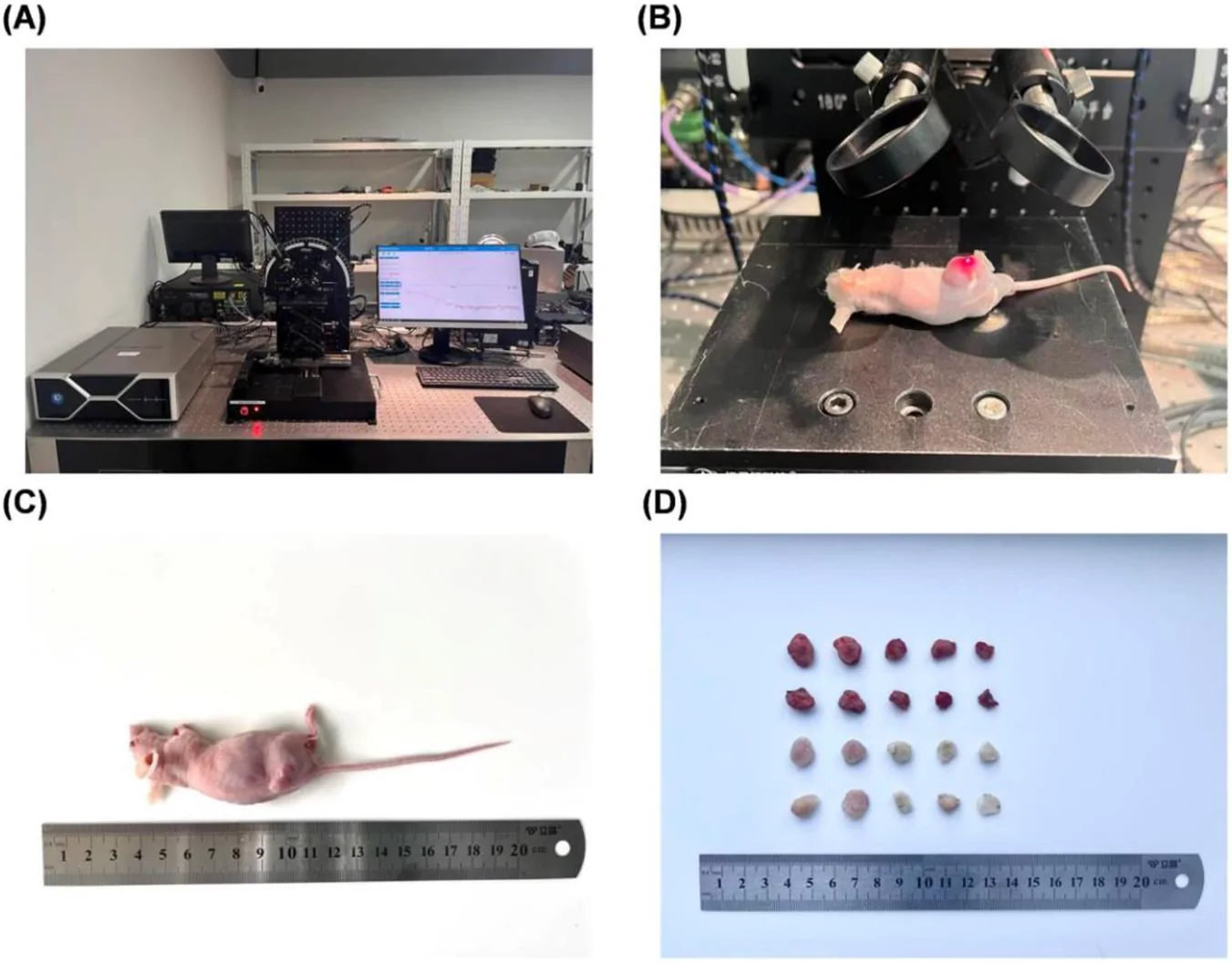
**(A)** Terahertz imaging system; **(B)** Terahertz imaging of a nude mouse; **(C)** Tumor-bearing nude mouse model; **(D)** Fresh tumor tissues: Upper panel, GNR-treated LUSC tissue; second panel, non-GNR-treated LUSC tissue; third panel, GNR-treated LUAD tissue; lower panel, non-GNR-treated LUAD tissue.

### 3.6 Preparation of tumor and lung tissue sections

Following imaging, tumor and lung tissues were sectioned and subjected to hematoxylin and eosin (H&E) staining for subsequent analysis. For nude mice, after anesthesia with 3% isoflurane inhalation, the mice were euthanized by cervical dislocation. Tumor tissues (Figure 2D) and lung tissues were harvested and fixed in 4% paraformaldehyde solution for 6 h. The tissues were then dehydrated using graded ethanol and xylene, and embedded in molten paraffin to form paraffin blocks. Tissue blocks were sectioned at a thickness of 10 μm using a microtome. Sections were mounted on both pathology-grade glass slides and high-density polyethylene (HDPE) slides for subsequent analysis. HDPE slides were prepared by cutting 1 mm thick HDPE sheets into 25 mm × 75 mm slides. Sections were affixed to HDPE slides to create HDPE-H&E sections for THz detection. Sections mounted on pathology-grade glass slides and stained with H&E were used for light microscopy to compare histological differences between healthy lung tissue and cancerous tissue. For human specimens, fresh tissue samples obtained after surgery were fixed in 10% formalin, processed routinely, and embedded in paraffin for storage as tissue blocks. Paraffin blocks of tumor tissue and adjacent normal tissue were collected, sectioned at a thickness of 5 μm using a microtome, and the specimen sections were subjected to H&E staining for microscopic examination and THz spectral measurements of unstained sections. The human-tissue arm intentionally evaluated compatibility with the routine pathology workflow (5 μm sections on glass) rather than providing a thickness- and substrate-matched comparison with the mouse sections; it should therefore be interpreted as a workflow-feasibility assessment.

### 3.7 THz-TDS scanning of H&E-stained sections

Transmission signals of tumor and normal tissue sections, denoted as Es(f) and Er(f), were measured using the THz-TDS system. These time-domain signals were subjected to fast Fourier transform to obtain the corresponding frequency-domain phase and amplitude spectra. Subsequently, the refractive index, absorption coefficient, and dielectric constant of the samples were calculated using the following equations:

$$n=\frac{\left[\psi \left(s\right)-\psi \left(r\right)\right]c}{2\pi fd}+1$$

$$\alpha =-\frac{2}{d}\ln\frac{\frac{A\left(s\right)}{A\left(r\right)}{\left(n+1\right)}^{2}}{4n}$$

(where n represents the refractive index, κ: the extinction coefficient, ε′: the real part of the dielectric constant, α: the absorption coefficient, c: the speed of light, d: the sample thickness, f: the frequency, s: the tumor group, and r: the lung tissue).

### 3.8 Microscopic observation of H&E-stained sections

Pathological sections of tumor tissues and lung tissues were placed under a microscope and observed at ×10 magnification to examine cellular structure and morphology.

## 4 Statistical analysis

Data were analyzed using SPSS 10.0. Spectra and images were visualized using Origin, MATLAB, CaseViewer, and Adobe Illustrator 2023. Continuous results are reported as mean ± standard deviation (SD). For image-based summaries, 50 pixels were sampled from each tumor region of interest (ROI) and 50 pixels from the adjacent skin/subcutaneous ROI; the resulting pixel-level SD describes within-image spatial variability and was not treated as biological replication. The mouse was the biological unit for *in vivo* comparisons, and the patient was the biological unit for human-tissue analyses. Two-group comparisons used the t-test, and comparisons among three groups used one-way analysis of variance (ANOVA); P < 0.05 was considered statistically significant. The contrast ratio (CR) was calculated as mean tumor-ROI signal divided by mean reference-ROI signal. Frequency-domain CRs were calculated separately at 0.5 and 0.9 THz.

## 5 Results

### 5.1 Cell experiments

After treating A549 and NCI-H520 cells with GNRs, cell proliferation was assessed using the CCK-8 assay and live/dead staining. Cells were cultured with GNR concentrations of 0, 20, 40, 60, 80, and 100 μg/mL. Figure 3A shows the mean reflected THz-field amplitude after removal of the culture medium. The amplitude increased with GNR concentration; the linear regression was Y = 0.197X + 4.26 (*R* ^2^ = 0.9871), where Y is THz reflectivity and X is GNR concentration. In the CCK-8 assay (Figure 3B), comparisons of A549 cells treated with 20, 40, or 60 μg/mL GNRs and NCI-H520 cells treated with 20 or 40 μg/mL GNRs with their respective controls were reported as P > 0.05. Live/dead images (Figures 3C–F) showed comparable staining patterns, and bright-field images showed no obvious morphological change after 40 μg/mL GNR treatment. These observations supported selection of 40 μg/mL for the subsequent experiments under the conditions tested.

**FIGURE 3:**
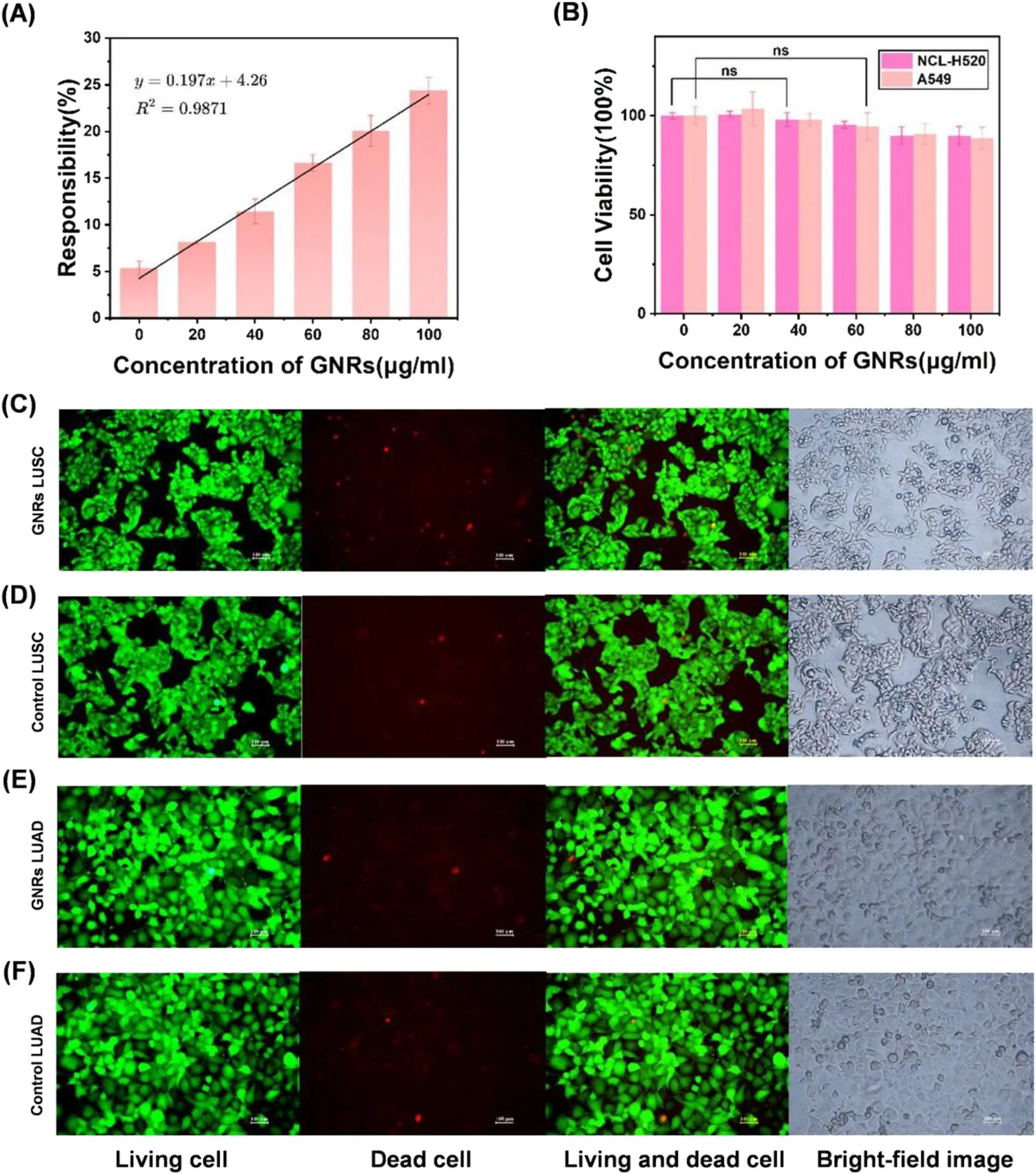
**(A)** THz reflectivity of cell preparations at 0, 20, 40, 60, 80, and 100 μg/mL GNRs; **(B)** CCK-8 cell-viability results for NCI-H520 and A549 cells at the indicated GNR concentrations (ns, not significant for the comparisons marked in the panel); **(C–F)** representative live/dead and bright-field images of GNR-treated and untreated NCI-H520 and A549 cells. Green indicates live cells and red indicates dead cells; scale bars are shown in the panels.

### 5.2 *In Vivo* THz imaging of tumor-bearing nude mice

Following successful model establishment (Figure 2C), tumor-bearing nude mice were anesthetized and subjected to whole-body scanning using a terahertz three-dimensional imaging system. THz-TDS measurements were performed at 25 °C with a relative humidity of 20%, a scanning step size of 200 μm, and a frequency range of 0.1− 3.0 THz (Figure 2A).

#### 5.2.1 Time-domain signal imaging

As shown in Figure 4, peak THz time-domain signal imaging revealed strong signal amplitude in the tumor region on the dorsal hind limb of nude mice, with signal intensity was higher than that of the adjacent background. The first row of images represents *in vivo* THz-TDS imaging of nude mice bearing GNR-treated NCI-H520 cell tumors (lung squamous cell carcinoma experimental group), while the second row represents imaging of nude mice bearing non-GNR-treated NCI-H520 cell tumors (lung squamous cell carcinoma control group). The third row represents *in vivo* THz-TDS imaging of nude mice bearing GNR-treated A549 cell tumors (lung adenocarcinoma experimental group), and the fourth row represents imaging of nude mice bearing non-GNR-treated A549 cell tumors (lung adenocarcinoma control group). The outlines of nude mice were delineated using Photoshop (PS) and superimposed on the terahertz images (solid red lines).

**FIGURE 4:**
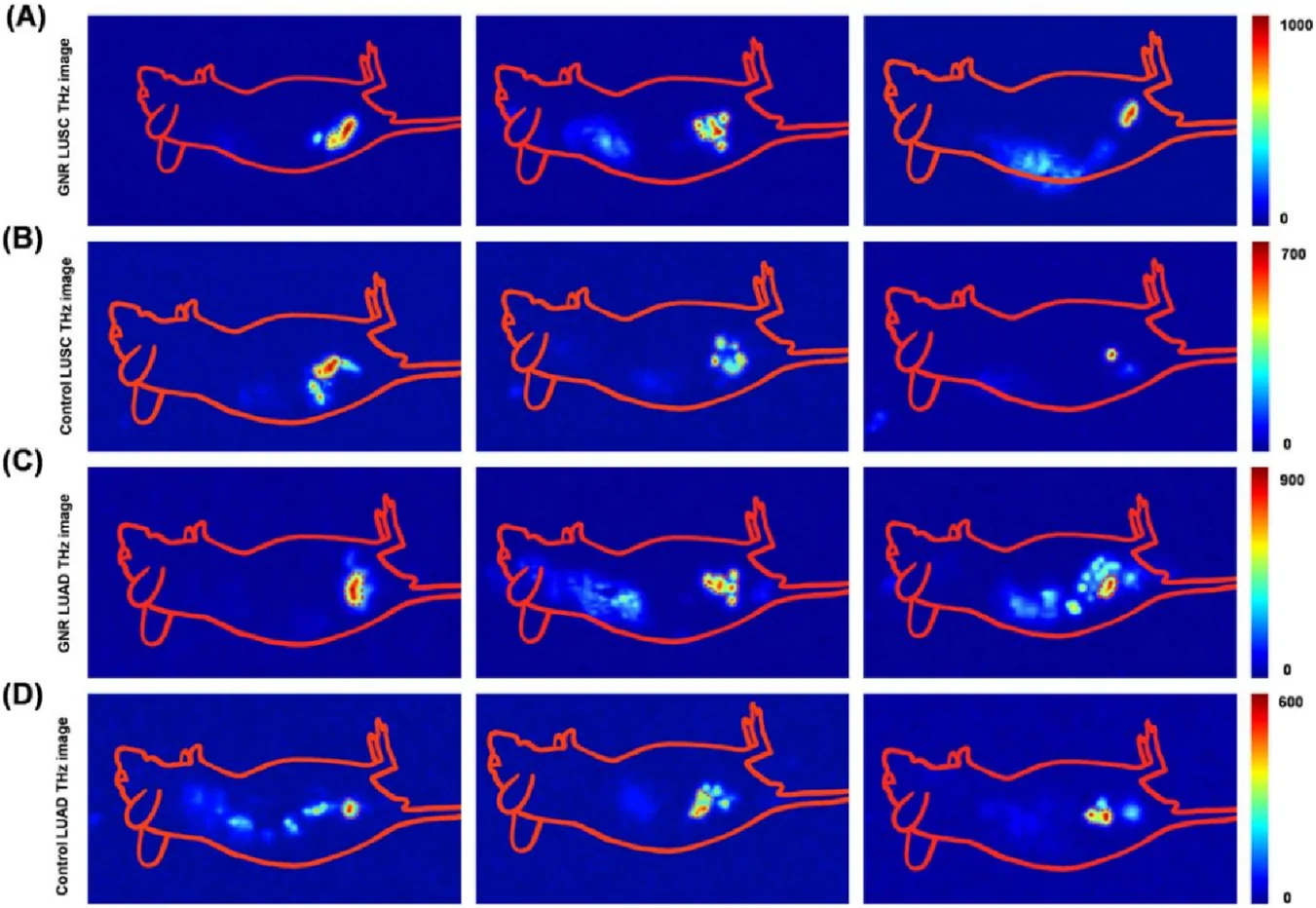
**(A)** Terahertz time-domain image of a nude mouse bearing GNR-treated LUSC tumors; **(B)** Terahertz time-domain image of a nude mouse bearing non-GNR-treated LUSC tumors; **(C)** Terahertz time-domain image of a nude mouse bearing GNR-treated LUAD tumors; **(D)** Terahertz time-domain image of a nude mouse bearing non-GNR-treated LUAD tumors.

For each image, 50 pixels were randomly sampled from the tumor ROI and 50 from the adjacent skin/subcutaneous ROI. The resulting per-image mean ± SD values describe within-image spatial variability. The tumor-center values for the three displayed GNR-treated NCI-H520 images were 1,111 ± 89, 954 ± 76, and 970 ± 81 A.U., compared with 664 ± 52, 617 ± 48, and 627 ± 50 A.U. for the displayed untreated NCI-H520 images. The corresponding values for GNR-treated A549 images were 754 ± 62, 783 ± 67, and 856 ± 71 A.U., compared with 522 ± 54, 495 ± 40, and 502 ± 45 A.U. for untreated A549 images. The adjacent skin/subcutaneous background was approximately 60 A.U. Across these displayed images, the mean tumor-region signal in each GNR-treated group was approximately 1.6-fold that of its untreated control.

Time-domain CR was calculated as mean tumor-ROI signal divided by mean adjacent skin/subcutaneous-ROI signal. The CRs were 17:1 and 11:1 for GNR-treated and untreated LUSC xenografts, respectively, and 13:1 and 8:1 for GNR-treated and untreated LUAD xenografts, respectively. These values represent tumor-to-adjacent-background contrast in a subcutaneous model and should not be interpreted as tumor-to-normal-lung CRs. Tumor outlines were superimposed on the live-animal images in Photoshop (red dashed lines) for visualization.

Time-domain data were also extracted along a 200-pixel horizontal line through the tumor region to generate the three-dimensional amplitude distributions in Figure 5. For the three displayed GNR-treated LUSC images, peak values were 1,193, 1,067, and 1061 A.U., compared with 771, 787, and 798 A.U. for untreated LUSC images. The corresponding GNR-treated LUAD peaks were 1,003, 967, and 834 A.U., compared with 680, 658, and 631 A.U. for untreated LUAD images. These descriptive values show higher amplitudes in the GNR-treated images and an approximately 1.6-fold increase in time-domain CR, rather than a twofold increase.

**FIGURE 5:**
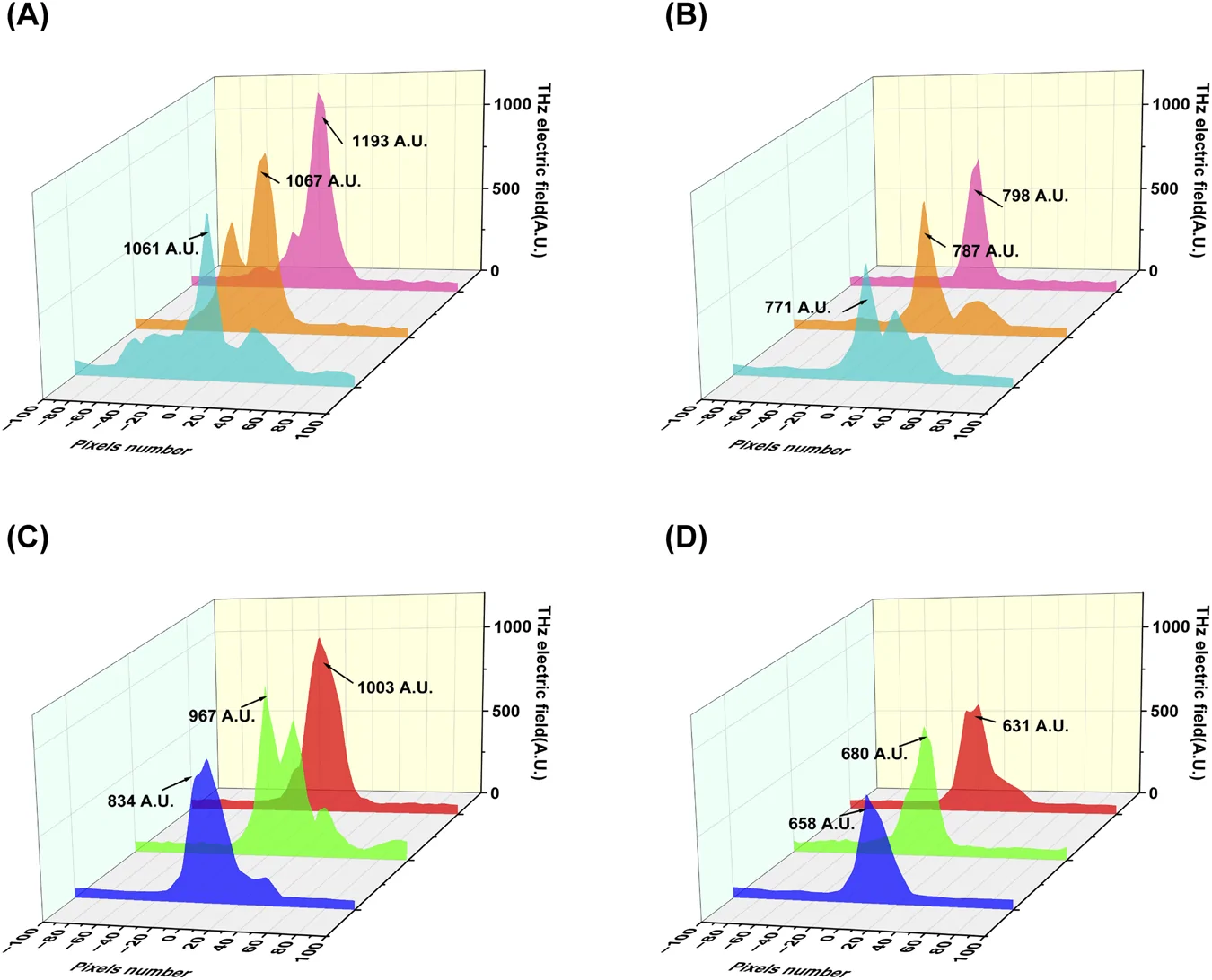
Three-dimensional electric field intensity distribution of 200 pixels in the tumor center for each group: **(A)** GNR-treated LUSC; **(B)** non-GNR-treated LUSC; **(C)** GNR-treated LUAD; **(D)** non-GNR-treated LUAD.

Subsequently, one point was selected from the tumor center region in each of the four groups of images to generate time-domain and frequency-domain waveform plots (Figure 6). As observed in Figures 6A,C, the peak time-domain signals in the experimental groups were higher than those in the control groups, with the control group peaks exhibiting a slight delay compared to the experimental groups. This delay may be attributed to the greater distance the THz signal traveled to reach the tumor surface. Examination of the THz frequency-domain data (Figures 6B,D) revealed markedly higher contrast in the experimental groups compared to both control groups and adjacent skin/subcutaneous background at 0.5 THz. At 0.9 THz, contrast remained higher in the experimental groups than in controls; however, beyond 1.3 THz, no consistent pattern in contrast was observed between the two groups, precluding effective imaging at these higher frequencies. These findings are consistent with the conclusions drawn from the imaging experiments presented in Figure 7.

**FIGURE 6:**
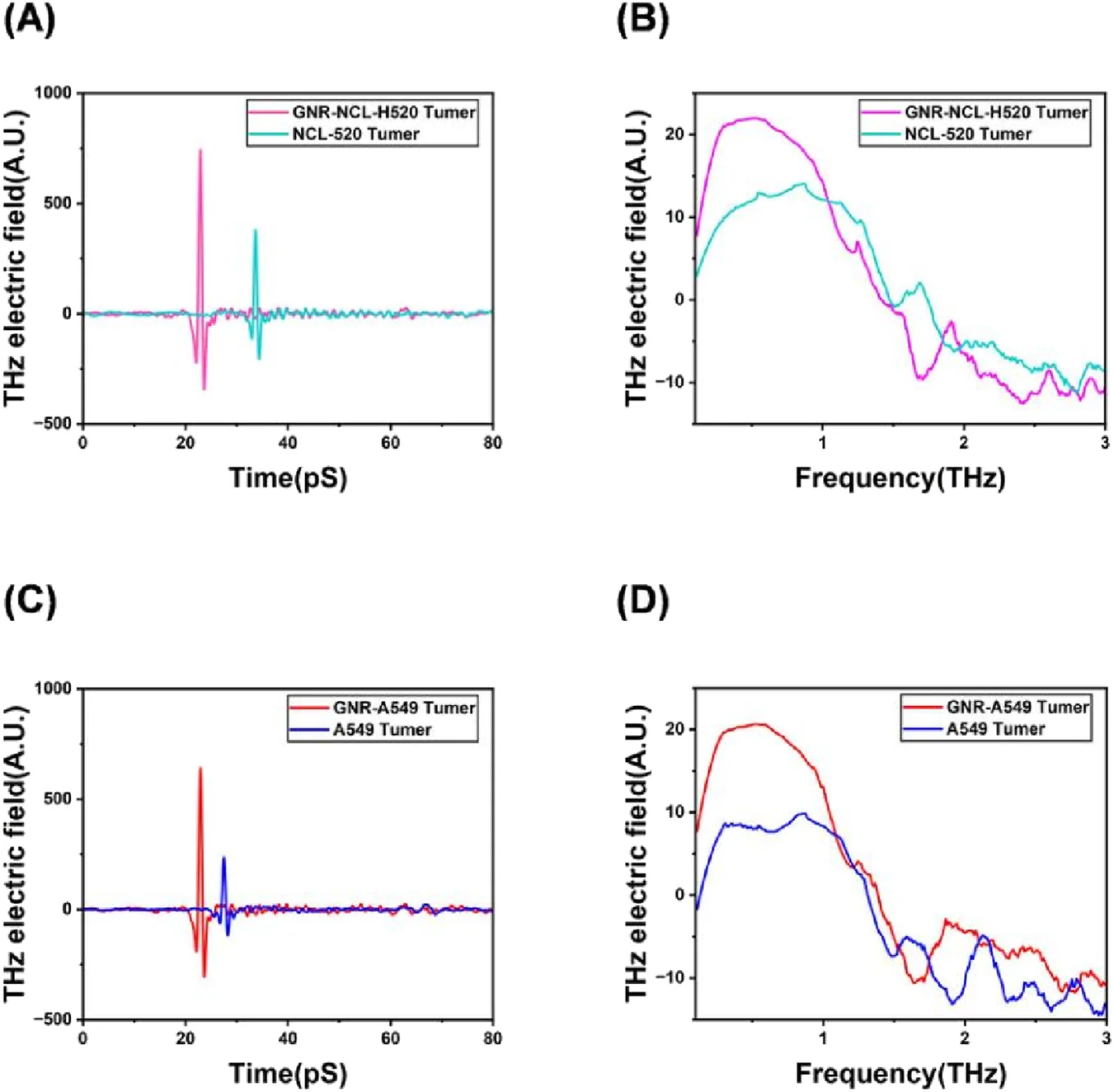
**(A)** Time-domain waveform of LUSC; **(B)** Frequency-domain waveform of LUSC; **(C)** Time-domain waveform of LUAD; **(D)** Frequency-domain waveform of LUAD.

**FIGURE 7:**
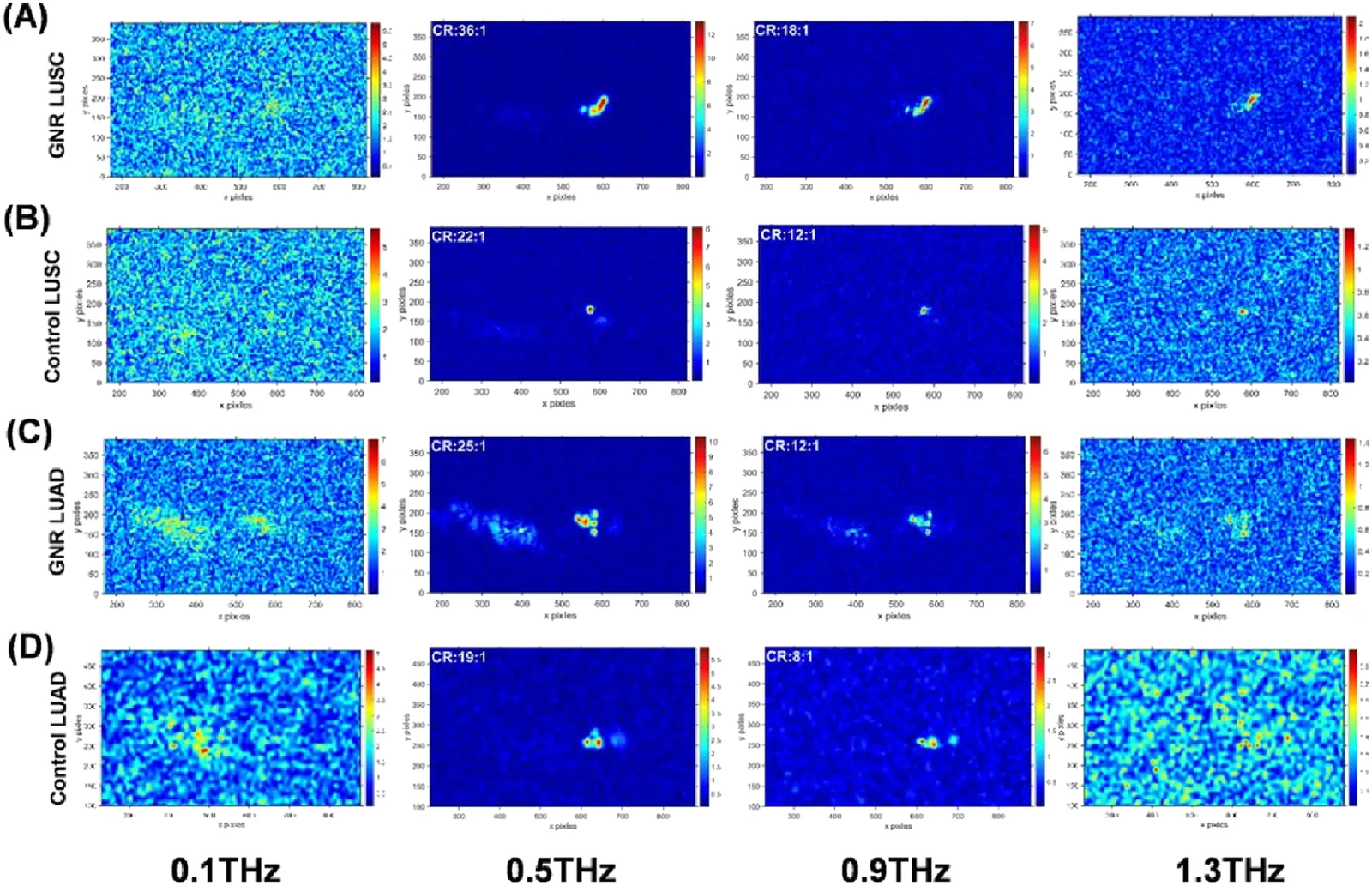
Frequency-domain THz images of **(A)** GNR-treated LUSC, **(B)** untreated LUSC, **(C)** GNR-treated LUAD, and **(D)** untreated LUAD xenografts. Columns show 0.1, 0.5, 0.9, and 1.3 THz. Each panel’s adjacent color bar gives THz electric-field amplitude in arbitrary units (A.U.; red = higher, blue = lower); numerical ranges differ by panel and are shown on the corresponding color bar. Images were acquired at 25 °C and 20% relative humidity with a 200 μm scanning step. CR labels are shown only where the ratio can be reproduced from the tabulated tumor and adjacent skin/subcutaneous ROI means.

The 0.5 THz wavelength is approximately 600 μm, far larger than an individual GNR. Therefore, the observed THz signal difference should not be attributed to direct resonance or Mie scattering by individual nanorods. Plausible, but unproven, indirect contributions include changes in the effective dielectric environment or hydration of the sampled tissue and scattering from tissue-scale heterogeneity or larger aggregates (Yan et al., 2020; Xu and Arbab, 2024; Brown et al., 2010). Because nanoparticle localization, aggregation, and tissue hydration were not measured directly, the present data do not distinguish among these mechanisms.

Figure 8 presents micro-CT images of the tumor-bearing mice in horizontal, coronal, and sagittal planes. CT served as an anatomical reference and localized the subcutaneous masses. Bone was readily visualized because of its high density, whereas the soft-tissue contrast between tumor, muscle, and fat was limited. The study was not designed to compare the diagnostic accuracy of CT and THz imaging; histopathology remained the reference standard for tissue classification.

**FIGURE 8:**
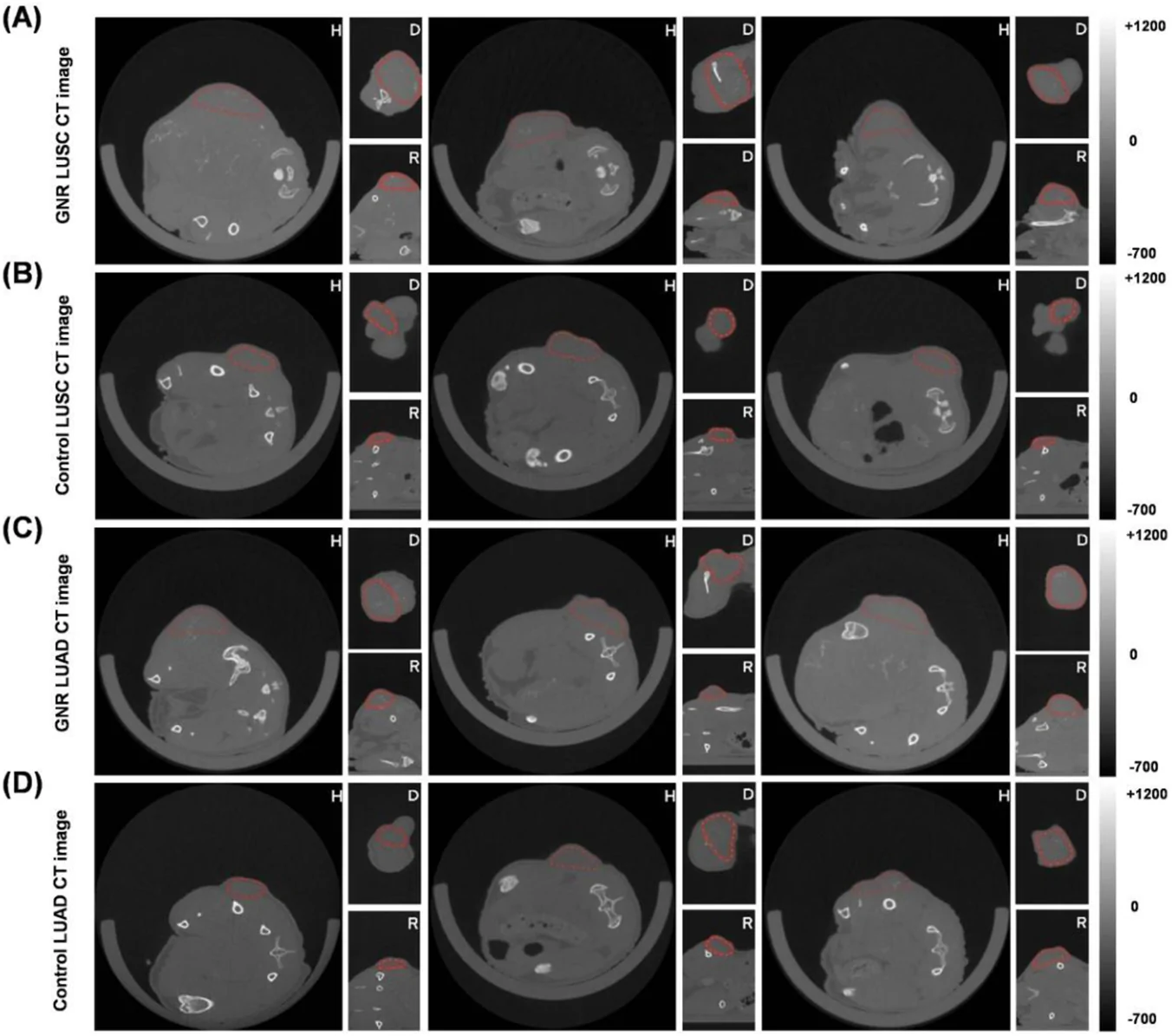
**(A)** CT image of the GNR-treated group with LUSC; **(B)** CT image of the non-GNR-treated group with LUSC; **(C)** CT image of the GNR-treated group with LUAD; **(D)** CT image of the non-GNR-treated group with LUAD.

#### 5.2.2 Frequency-domain signal imaging

Following THz time-domain spectroscopy imaging, *in vivo* imaging of tumor-bearing nude mice was performed using THz frequency-domain spectroscopy. As shown in Figure 7, the THz signals were subjected to fast Fourier transform (FFT) to generate frequency-domain images of mouse tumors within the 0.1–3 THz range. *In vivo* THz imaging was conducted at frequencies of 0.1, 0.5, 0.9, and 1.3 THz for both experimental and control groups. Multiple points were selected along the tumor-healthy tissue boundary for reflective single-point scanning.

At 0.5 and 0.9 THz, tumor-region field amplitudes were higher in the GNR-treated groups than in the corresponding untreated groups, and the LUSC groups showed higher amplitudes than the LUAD groups. THz amplitude data were extracted from tumor ROIs and adjacent skin/subcutaneous ROIs. CR was calculated as the tumor-ROI mean divided by the reference-ROI mean and was evaluated separately at 0.5 and 0.9 THz.

At 0.5 THz, the GNR-treated LUSC group had mean amplitudes of 12.0 A.U. in the tumor ROI and 0.33 A.U. in the adjacent skin/subcutaneous ROI, giving a CR of 36:1. The untreated LUSC group had corresponding means of 5.86 and 0.27 A.U., giving a CR of 22:1; the treated-to-untreated CR ratio was therefore approximately 1.7. At 0.9 THz, the GNR-treated LUSC means were 6.62 and 0.36 A.U. (CR 18:1), whereas the untreated means were 3.24 and 0.24 A.U. (CR 12:1). At 0.5 THz, the GNR-treated LUAD group exhibited mean amplitudes of 9.25 A.U. in the tumor ROI and 0.36 A.U. in the adjacent skin/subcutaneous ROI, yielding a CR value of 25:1; the untreated LUSC group showed corresponding mean values of 4.28 and 0.23 A.U., with a CR value of 19:1. At 0.9 THz, the GNR-treated LUSC group had mean values of 4.22 and 0.35 A.U. (CR = 12:1), while the untreated group showed mean values of 2.62 and 0.33 A.U. (CR = 8:1).

In the subcutaneous xenograft model, 0.5 THz provided the clearest images among the tested bands. At 0.9 THz, the GNR-treated groups also showed higher descriptive CRs than their untreated controls. At 0.1 and 1.3 THz, no consistent enhancement was observed, and differentiation between tumor and adjacent background was limited.

## 6 Terahertz imaging of paraffin-embedded tissues

After *in vivo* imaging, subcutaneous tumors were harvested, dehydrated, and embedded in paraffin. THz-TDS measurements were performed at 25 °C, 20% relative humidity, a 200 μm scanning step, and 0.1–3.0 THz. As shown in Figure 9, for the three displayed samples per group, mean ± within-image SD values from 50 tumor-ROI pixels were: GNR-treated LUSC, 1,278 ± 86, 1,219 ± 93, and 1,226 ± 97 A.U (Figure 9A); untreated LUSC, 1,163 ± 90, 1,124 ± 82, and 1,087 ± 88 A.U (Figure 9B); GNR-treated LUAD, 1,129 ± 98, 1,103 ± 90, and 1,152 ± 91 A.U (Figure 9C); and untreated LUAD, 1,052 ± 79, 1,009 ± 83, and 1,034 ± 82 A.U (Figure 9D). The surrounding paraffin signal was approximately 700 A.U., and descriptive CRs were approximately 2:1. The four-group comparison was reported as P > 0.05. The reduced separation is consistent with loss of hydration-based contrast and the change to a paraffin background (Brown et al., 2010). GNR redistribution or loss during processing is also possible, but GNR retention was not measured; these mechanisms cannot be distinguished by the present data.

**FIGURE 9:**
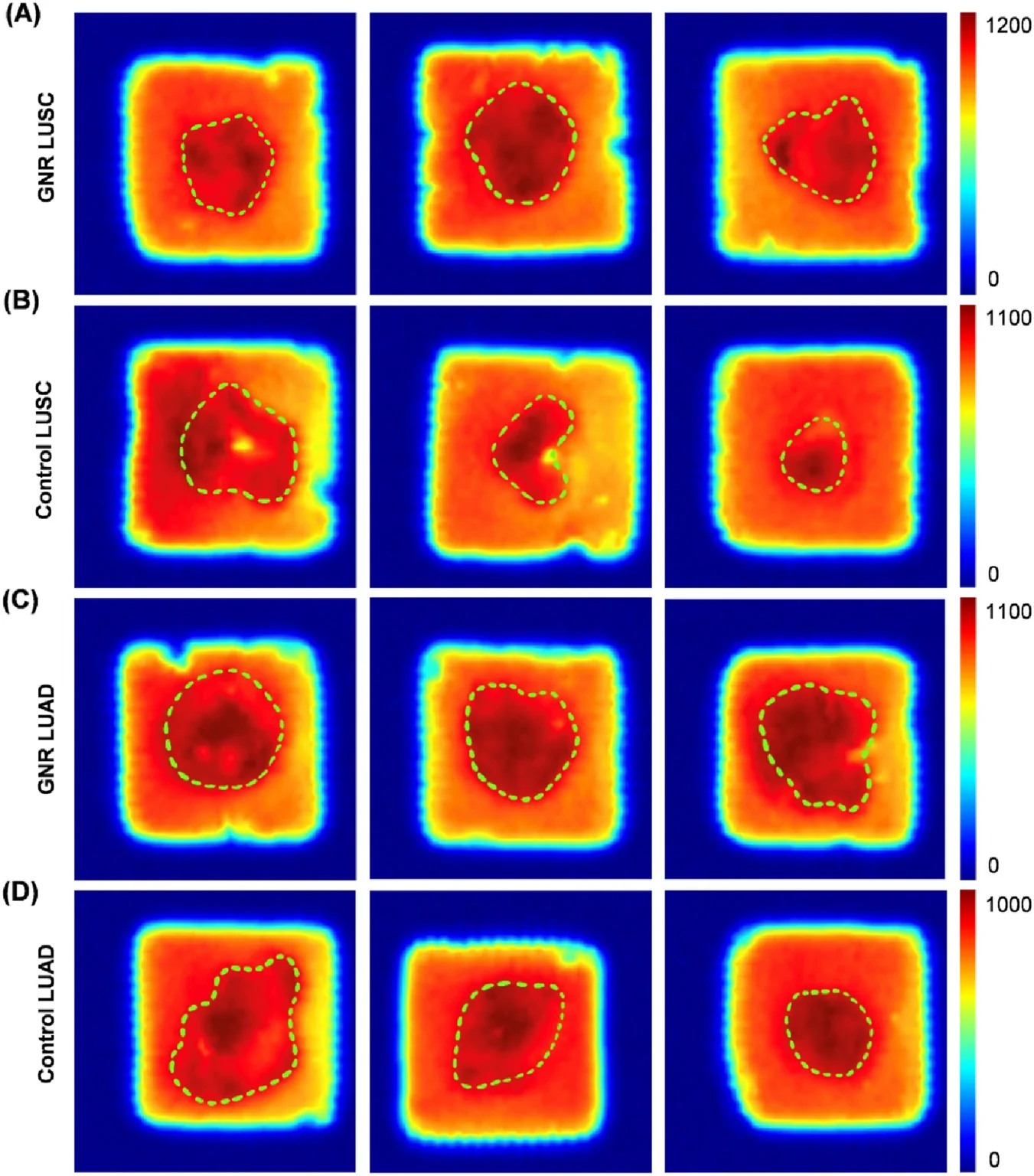
**(A)** Terahertz time-domain image of paraffin-embedded GNR-treated LUSC tissue; **(B)** Terahertz time-domain image of paraffin-embedded non-GNR-treated LUSC tissue; **(C)** Terahertz time-domain image of paraffin-embedded GNR-treated LUAD tissue; **(D)** Terahertz time-domain image of paraffin-embedded non-GNR-treated LUAD tissue.

### 7 THz-TDS analysis of H&E-stained sections from lung cancer and normal tissues

#### 7.1 THz-TDS characteristics of nude mouse tissues

In mouse sections (Figure 10), refractive-index and absorption-coefficient curves differed among GNR-treated tumor, untreated tumor, and normal lung tissue. For LUSC, one-way ANOVA indicated group differences in refractive index and absorption coefficient (P < 0.05); the GNR-treated LUSC group reached a peak absorption coefficient of approximately 90 cm^-1^ near 1.5 THz. LUAD showed similar curve shapes, with a peak absorption coefficient of approximately 85 cm^-1^. One-way ANOVA likewise indicated group differences in LUAD refractive index and absorption coefficient (P < 0.05). These thresholded P statements summarize the available analysis and should not be interpreted as evidence for a specific nanoparticle-localization mechanism.

**FIGURE 10:**
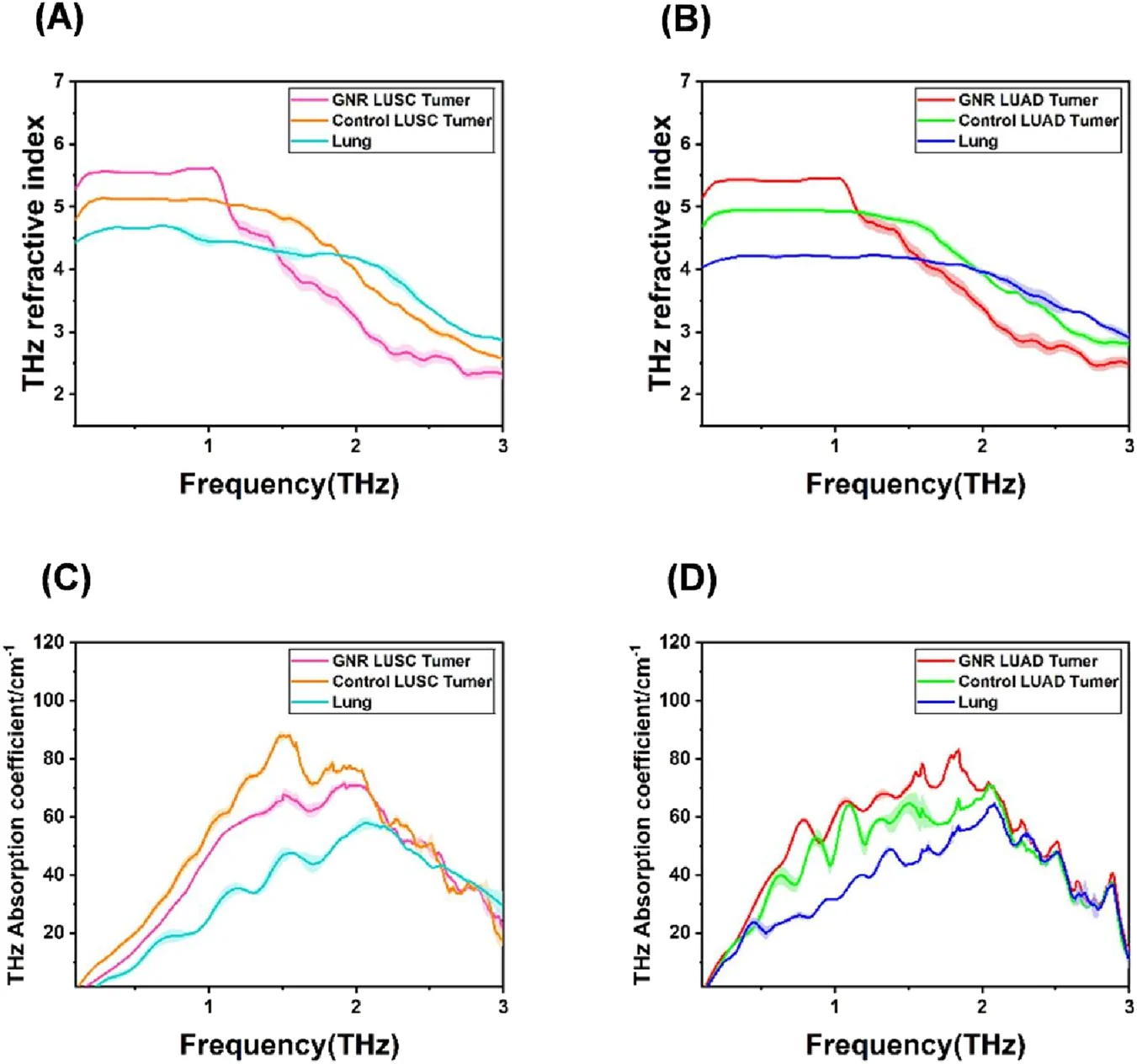
**(A)** Refractive index of LUSC in nude mice; **(B)** Refractive index of LUAD in nude mice; **(C)** Absorption coefficient of LUSC in nude mice; **(D)** Absorption coefficient of LUAD in nude mice.

### 7.2 THz-TDS characteristics of human tissues

THz spectroscopy was performed on sections from 9 LUSC cases and 9 LUAD cases, together with adjacent normal lung tissue (Figure 11). Mean refractive indices of LUSC and LUAD were descriptively higher than those of normal lung tissue, significant differences among the three groups were detected below approximately 1.2 THz (P < 0.05). Near the absorption-coefficient peak, LUSC and LUAD values were slightly higher than normal lung values, but the tumor-versus-normal comparisons were also not significant (P > 0.05). These negative findings may reflect the weak signal from 5 μm sections and interference from pathology-grade glass; they do not establish absence of intrinsic biological differences.

**FIGURE 11:**
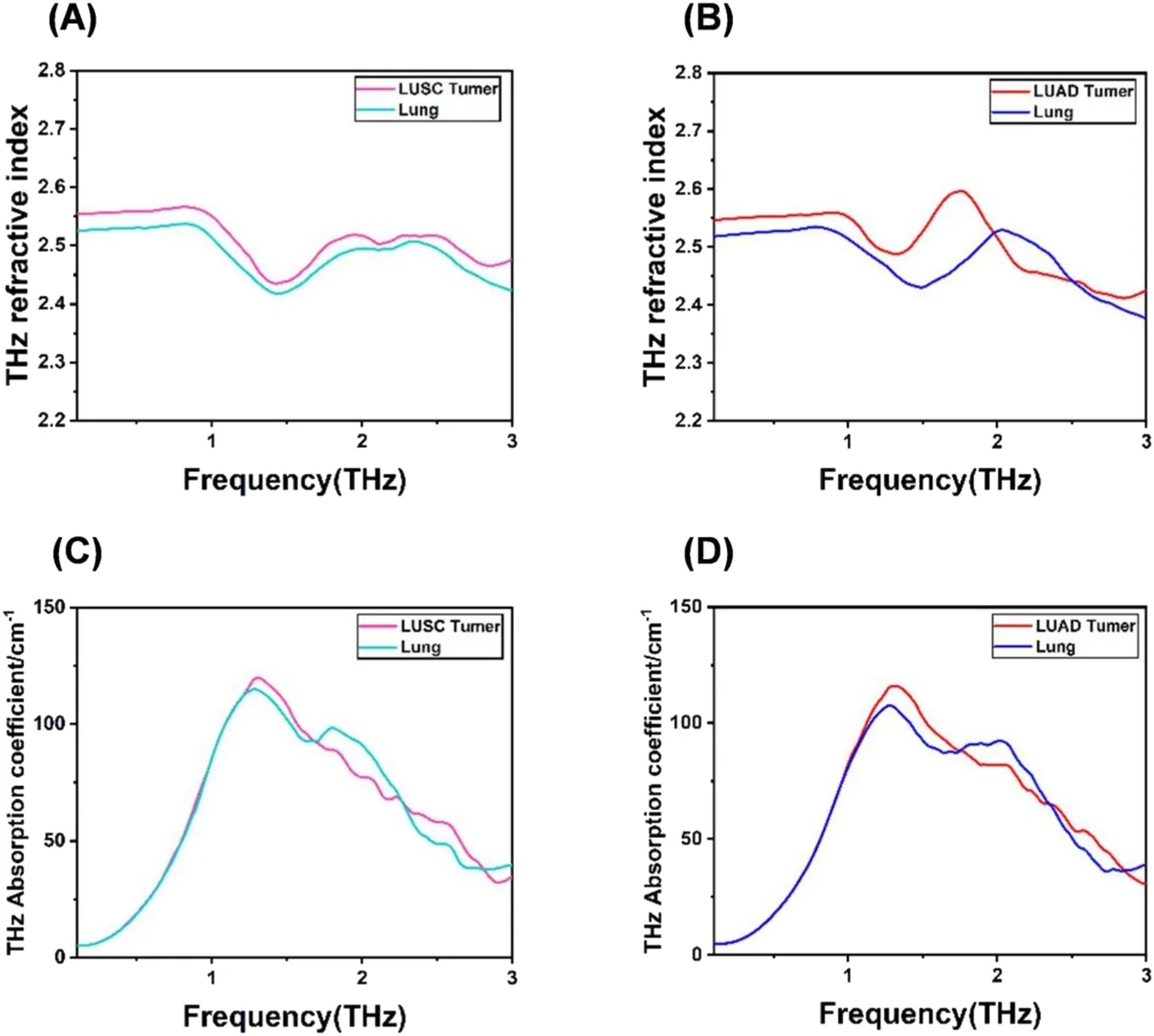
**(A)** Refractive index of human LUSC; **(B)** Refractive index of human LUAD; **(C)** Absorption coefficient of human LUSC; **(D)** Absorption coefficient of human LUAD.

### 8 Histopathological features

As shown in Figure 12, panel A shows an HE-stained pathological section of lung squamous cell carcinoma; panel B shows an HE-stained pathological section of lung adenocarcinoma; and panel C shows an HE-stained pathological section of normal lung tissue. As shown in Figure 12D, H&E-stained sections of lung squamous cell carcinoma revealed tumor cells arranged in nests or sheets, exhibiting a marked tendency toward keratinization, with keratin pearl formation observed in some areas. The nuclei displayed pleomorphism, hyperchromatic chromatin, prominent nucleoli, and readily identifiable mitotic figures. The tumor tissue showed indistinct boundaries with the surrounding stroma, demonstrating infiltrative growth, often accompanied by necrosis and inflammatory cell infiltration. These features reflect the typical morphology of epithelial malignancies originating from squamous cells, with their tight cellular junctions and abundant keratin components constituting a unique tissue microenvironment (Xin et al., 2025).

**FIGURE 12:**
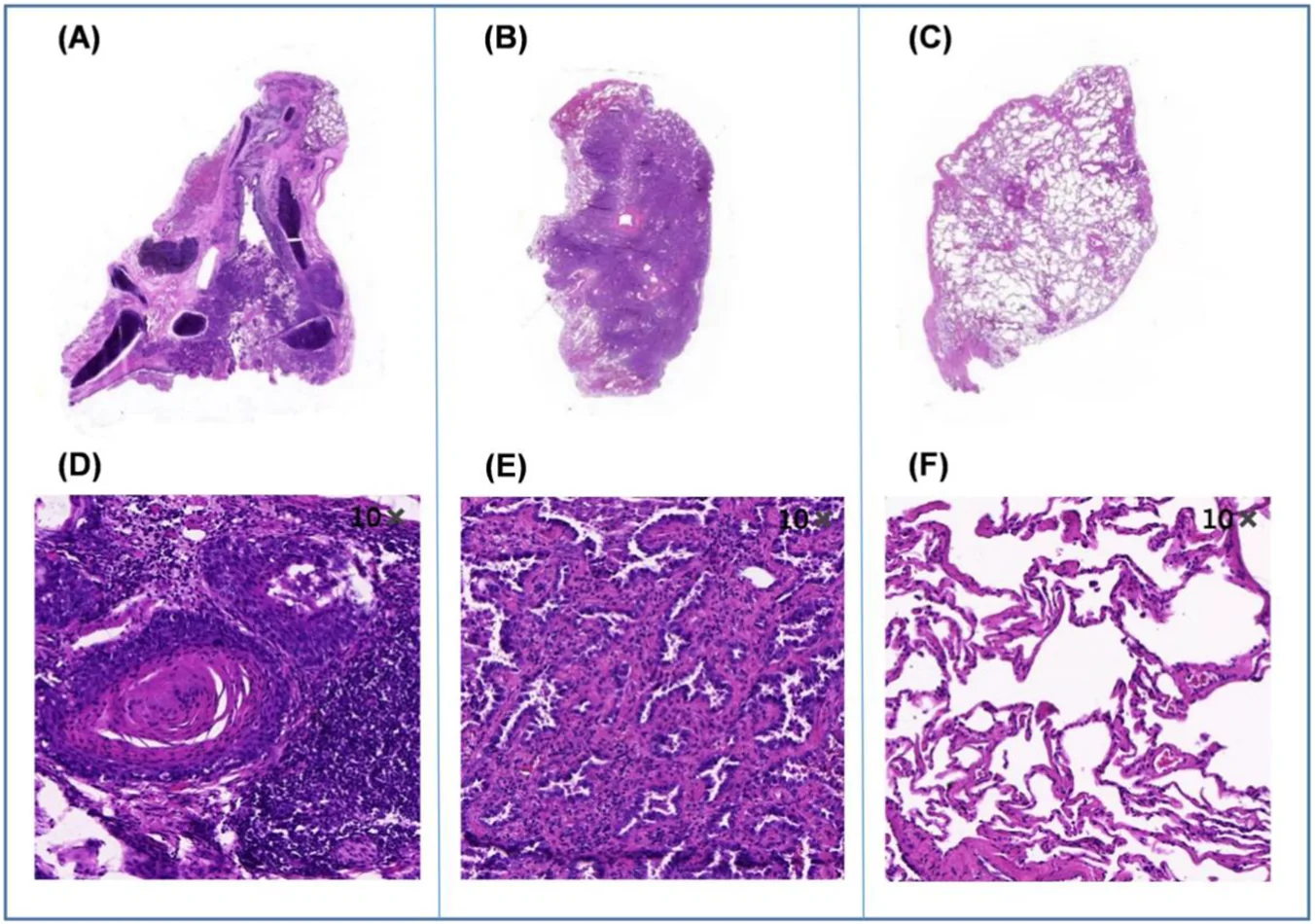
**(A)** Pathological section of lung squamous cell carcinoma; **(B)** Pathological section of lung adenocarcinoma; **(C)** Pathological section of normal lung tissue; **(D)** Magnified view (×10) of **(A,E)** Magnified view (×10) of **(B,F)** Magnified view (×10) of **(C)**.

In contrast, as shown in Figure 12E, lung adenocarcinoma tissue exhibited distinct histological morphology. Tumor cells formed acinar, papillary, or solid structures, with cuboidal or columnar morphology and frequently abundant cytoplasm. Nuclear atypia was pronounced, with prominent nucleoli and active mitotic figures. The structural characteristics of adenocarcinoma are closely associated with its secretory function and glandular differentiation (Shambayati and Evered, 2023).

Normal lung tissue (Figure 12F) displayed a completely different, highly organized structure. Alveolar structures were intact, with thin alveolar walls composed of epithelial cells, and capillary networks closely surrounding the alveoli to facilitate efficient gas exchange. Bronchioles exhibited regular lumina, with this highly specialized architecture maximizing ventilation and gas exchange function (Hon and Naidu, 2025).

The histological differences among LUSC, LUAD, and normal lung may contribute to variation in their THz optical properties. Compact cellularity and keratin-rich structures in LUSC, glandular architecture and mucus in LUAD, and air-rich alveolar spaces in normal lung could influence local water distribution, effective refractive index, and scattering. However, water content, macromolecular concentration, and morphological parameters were not quantified in this study; the proposed links to THz signal differences therefore remain hypotheses rather than demonstrated causal explanations.

Tissue cell density, nuclear-to-cytoplasmic ratio, biomolecular composition, extracellular matrix, and water distribution are plausible contributors to THz wave-tissue interactions (Rytik and Tuchin, 2025). The observed differences in refractive index, absorption coefficient, and time-domain pulse behavior are consistent with differences in pathological morphology and composition, but the present data do not establish a quantitative causal relationship. Future studies should prospectively measure these morphological and compositional variables and test their associations with THz optical parameters.

## 9 Discussion

This study provides proof-of-concept evidence that GNR treatment is associated with higher reflected THz amplitude in subcutaneous LUSC and LUAD xenografts. The findings should be interpreted descriptively because the study did not directly establish nanoparticle localization or the physical origin of the contrast.

- Mechanistic interpretation. The reported GNR localized surface plasmon resonance near 800 nm corresponds to approximately 375 THz and lies far above the 0.1–3.0 THz detection band; Thus, the real part of the dielectric constant becomes strongly negative according to Drude model, and the THz waves cannot propagate inside but reflected. Here, the GNRs with high carrier density behave like a perfect metallic “mirror” at the experimental frequency, which is a very classic phenomena in the THz regime. (THz-TDS, 2011). Finally, the reflected THz signals get enhanced compared with the non GNR-treated samples.

Plausible indirect contributions include changes in the effective dielectric environment or hydration of the sampled tissue and scattering from tissue-scale heterogeneity or larger aggregates (Xu and Arbab, 2024; Brown et al., 2010). At 0.5 THz, the wavelength is approximately 600 μm, much larger than an individual GNR, so individual-nanorod Mie scattering should not be invoked. The study did not include dielectric-particle controls, a controlled dehydration series, TEM or dark-field localization, or quantitative measurements of GNR retention after processing. The mechanism, localization, and aggregation state therefore remain unresolved.

II. Tissue context and pathological interpretation. Histological differences between LUSC, LUAD, and normal lung may contribute to the observed THz patterns. Compact cellularity and keratinization in LUSC, glandular architecture and mucus in LUAD, and air-rich alveolar structure in normal lung could alter water distribution, effective refractive index, and scattering. However, these relationships were not quantified, and they should be considered hypotheses rather than demonstrated causal explanations. Future studies should correlate pre-specified morphological measures with THz optical parameters.
III. Effects of paraffin processing. Contrast decreased markedly after fixation, dehydration, and paraffin embedding. Removal of water changes the dominant THz contrast, while paraffin introduces a different dielectric background (Brown et al., 2010). GNR redistribution or loss during processing may also contribute, but GNR retention was not measured. The current data therefore cannot determine whether reduced contrast primarily reflects dehydration, paraffin masking, nanoparticle loss, or a combination of these factors.
IV. Human-section workflow. The human-tissue arm used routine 5 μm sections on pathology-grade glass to test compatibility with an existing clinical workflow, whereas the mouse sections used a THz-optimized 10 μm/HDPE configuration. This was not a thickness- and substrate-matched comparison. The weak human-section separation may therefore reflect substrate and thickness constraints and should not be used to infer equivalence between human tumor and normal tissue. Matched comparisons on THz-transparent substrates are required.
V. Translational scope and roadmap. The subcutaneous xenograft model differs from orthotopic lung tumors in alveolar architecture, vascular pulsation, pleural context, and stromal composition. Moreover, the reported *in vivo* CR uses adjacent skin/subcutaneous tissue, not normal lung, as the reference. Clinical extrapolation must therefore remain cautious. Translation would require compact reflection/contact probes, Qingdao Qingyuan Fengda Technology Co., Ltd. Is launching a contact-type portable terahertz time-domain spectrometer. controlled humidity, faster or gated acquisition with motion correction, strategies to limit or account for blood and surgical-fluid attenuation, and validation in orthotopic models and prospective fresh human specimens. These are future engineering and validation requirements; the present system remains at the proof-of-concept stage and is not positioned to replace routine histopathology.

In conclusion, GNR treatment was associated with higher THz signals in subcutaneous LUSC and LUAD xenografts under the tested conditions, with the clearest image contrast at 0.5 THz. Because the mechanism, nanoparticle localization, orthotopic performance, and robustness in blood- or fluid-rich surgical fields were not established, these findings support a proof-of-concept rather than clinical readiness. At present, the approach is most plausibly applicable to fresh or sufficiently thick hydrated tissue and should be viewed as complementary to, not a replacement for, standard histopathology.

## Data Availability

The original contributions presented in the study are included in the article/supplementary material, further inquiries can be directed to the corresponding authors.
